# Geographical variation and factors influencing modern contraceptive use among married women in Ethiopia: evidence from a national population based survey

**DOI:** 10.1186/1742-4755-10-52

**Published:** 2013-09-26

**Authors:** Yihunie Lakew, Ayalu A Reda, Habtamu Tamene, Susan Benedict, Kebede Deribe

**Affiliations:** 1 Ethiopian Public Health Association, Addis Ababa, Ethiopia; 2 Global Health, Brown Advanced Research Institutes, Population Studies and Training Center, Brown University, Providence, Rhode Island, United States of America; 3 Population Service International in Ethiopia, Addis Ababa, Ethiopia; 4 University of Texas School of Nursing, Houston, Texas, United States of America; 5 Brighton and Sussex Medical School, Falmer, Brighton, United Kingdom; 6 Addis Ababa University School of Public Health, Addis Ababa, Ethiopia

## Abstract

**Background:**

Modern contraceptive use persists to be low in most African countries where fertility, population growth, and unmet need for family planning are high. Though there is an evidence of increased overall contraceptive prevalence, a substantial effort remains behind in Ethiopia. This study aimed to identify factors associated with modern contraceptive use and to examine its geographical variations among 15–49 married women in Ethiopia.

**Methods:**

We conducted secondary analysis of 10,204 reproductive age women included in the 2011 Ethiopia Demographic and Health Survey (DHS). The survey sample was designed to provide national, urban/rural, and regional representative estimates for key health and demographic indicators. The sample was selected using a two-stage stratified sampling process. Bivariate and multivariate logistic regressions were applied to determine the prevalence of modern contraceptive use and associated factors in Ethiopia.

**Results:**

Being wealthy, more educated, being employed, higher number of living children, being in a monogamous relationship, attending community conversation, being visited by health worker at home strongly predicted use of modern contraception. While living in rural areas, older age, being in polygamous relationship, and witnessing one’s own child’s death were found negatively influence modern contraceptive use. The spatial analysis of contraceptive use revealed that the central and southwestern parts of the country had higher prevalence of modern contraceptive use than that of the eastern and western parts.

**Conclusion:**

The findings indicate significant socio-economic, urban–rural and regional variation in modern contraceptive use among reproductive age women in Ethiopia. Strengthening community conversation programs and female education should be given top priority.

## Introduction

Globally, each year, nearly 350,000 women die while another 50 million suffer illness and disability from complications of pregnancy and child birth [1]. It has been reported that Ethiopia is one of among six countries that contribute to about 50% of the maternal deaths along with India, Nigeria, Pakistan, Afghanistan and the Democratic Republic of Congo [1]. The Ethiopia Demographic Health Surveys of 2000, 2005 and 2011 gave figures of 871, 673, 676 per 100,000 live births maternal mortality ratios respectively [2-4].

Modern family planning methods are widely believed to influence fertility reduction worldwide [5]. Family planning had a clear effect on the health of women, children, and families worldwide – especially those in developing countries [6]. Globally, contraceptives help to prevent an estimated 2.7 million infant deaths and the loss of 60 million of healthy life in a year [6]. Promotion of family planning in countries with high birth rates has the potential to reduce poverty and hunger and avert 32% of all maternal deaths and nearly 10% of childhood deaths [7]. It would also contribute substantially to women’s empowerment, achievement of universal primary schooling, and long-term environmental sustainability. In the past few decades, family-planning programs have played a major part in raising the prevalence of contraceptive practice from less than 10% to 60% and reducing fertility in developing countries from six to about three births per woman [7].

The modern family planning service in Ethiopia started in 1966 [8] but showed little signs of expansion for an extended period of time. However, in the last 20 years, with the adoption of the population policy in 1993 [9,10], numerous local and international partners in family planning have come together to assist the government in expanding family planning programs and services. The National Population Office was established to implement and oversee the strategies and actions related to the population policy [8]. In 1996, the Ministry of Health released Guidelines for Family Planning Services in Ethiopia to guide health providers and managers, as well as to expand and ensure quality family planning services in the country [11]. The ministry designed new outlets for family planning services in the form of community-based distribution, social marketing, and work-based services, in addition to the pre-existing facility-based and outreach family planning services. Work-based services are services made available to users at their place of work such as factories, prisons, and schools [8]. Moreover, in the last decade, integration and linkage between family planning services and HIV/AIDS care, along with maternal and other reproductive health services, have been emphasized in guidelines and strategic documents with the aim of enhancing family planning utilization [8]. Currently, the service has been provided to rural communities at the household level through the Health Extension Programme. Moreover, in the current road map for accelerating the reduction of maternal and newborn morbidity and mortality in Ethiopia (2011–2015), family planning is identified as one of the strategic objectives. The following targets are identified related to family planning: to increase contraceptive prevalence rate to 66%, decrease unmet needs for family planning to 10%, and reduce adolescent pregnancy rate to 5% [12].

Though the overall contraceptive prevalence has been progressive with evidences of 2.6%, 8%, 14%, and 29% reported in 1990, 2000, 2005 and 2011 respectively [2-4,13]. The use of modern contraceptive method differs significantly among regions, urban and rural areas. The main objective of this paper is to examine factors associated with contraceptive usage and spatial distributions of contraceptive use among married women.

## Methods

### Study settings and sampling

Ethiopia is administratively divided into nine regional states and two city administrations. These are subdivided into 817 administrative Woredas (districts) which are further divided into around 16,253 Kebeles, the smallest administrative units in the administrative structure of the country. According to the projections of the 2007 population and housing census, the total population of the country for the year 2010 was estimated to be 79.8 million [14]. Close to 80% of the Ethiopian population lives in rural areas. The average size of a household is 4.6 individuals. The fertility trend in recent years shows that there has been a marked decline in the total fertility rate from the 1990 level of 6.4 births to 4.8 births per woman in 2011 [2].

The 2011 Ethiopia Demographic and Health Survey (2011 EDHS) which we analyze in this study is the third DHS in Ethiopia [2]. The sample for the 2011 EDHS was designed to provide population and health indicators at the national and regional levels. The sample was selected using a stratified two-stage cluster sampling design. This design allowed for specific indicators, such as contraceptive use, to be calculated for each of Ethiopia’s 11 geographic/administrative regions (the nine regional states and two city administrations). The 2007 Population and Housing Census, conducted by the central statistical agency (CSA), provided the sampling frame from which the 2011 EDHS sample was drawn [2,14]. The sample for the survey was designed to represent national, urban–rural, and regional estimates of health and demographic outcomes. In the first stage, 624 clusters of census enumeration areas, 187 in urban areas, and 437 in rural areas were included in the survey. In the second stage, a complete listing of households was carried out in each of the 624 selected EAs from September 2010 through January 2011. Sketch maps were drawn for each of the clusters, and all conventional households were listed. A representative sample of 17,817 households was selected for the 2011 EDHS. Subsequently a total of 16,515 women in the age group 15–49 years who were usual residents or who slept in the selected households the night before the survey were eligible and interviewed for the survey [2].

### Survey instrument and data extraction

The DHS questionnaires were adapted from model survey instruments developed for the MEASURE DHS project to reflect the population and health issues relevant to Ethiopia. The adaptation of the questionnaire was conducted through a series of meetings with the various stakeholders. In addition to the English language, the questionnaires were translated into three major local languages—Amharigna, Oromiffa, and Tigrigna. The Woman’s Questionnaire was used to collect information from all women of reproductive age (15–49 years). We downloaded the women public access DHS dataset in SPSS format. Further data cleaning was done by the investigators. Data on a total of 10,204 married women of reproductive age were included in the analysis. Information on a wide-range of potential independent variables (socio-demographic, economic, fertility history, etc.) was extracted accordingly.

### Outcome measures

The use of modern contraception was analyzed for married women aged 15–49 years who reported that they were currently on modern contraceptive use. Modern contraceptive use refers to a measure of whether a woman was using a modern method of contraception (oral pill, intrauterine device, condom, female or male sterilization, implant, or injectable) at the time of the survey. Dummy variables are created for this variable for which use of modern contraceptives was assigned “yes” (coded as 1) and not using modern contraceptive was coded as “No” (coded as 0).

### Exposure measures

Potential predictors modern contraceptive use such as age, household size, occupation, child mortality, parity, religion, women’s education, marital status, husband’s education, and wealth index were included in the analysis. Community-level variables included in the analysis were place of residence (urban, rural) and region.

### Data analysis

Survey weights provided by the DHS were used to compute the prevalence of modern contraceptive use. Binary logistic regression was performed to explore association between the dependent variable and a wide range of independent variables. P-values of less than 0.05 were considered as statistically significant. Potential independent variables were entered simultaneously in the model in order to examine the net effect of each variable.

The prevalence data were exported into ArcGIS to visualize key estimations. The cluster levels of modern contraceptive prevalence rates were used to develop prevalence maps at zonal, regional, and cluster levels in ArcGIS software. Spatial heterogeneity of high prevalence/low prevalence areas of modern contraception use was examined using the Getis-Ord G-statistic and associated Z-scores were computed for each cluster in ArcGIS 10 (ESRI Inc. USA) using the Spatial Statistics tool. A high or low value of the G-statistic indicates that high/low values prevalence were clustered within the study area. To determine the significance of these statistics, Z-scores were used. A z-score near zero indicates no apparent clustering within the study area. A positive z-score indicates clustering of high values. A negative z-score indicates clustering of low values.

### Ethical clearance

The study is based on secondary analysis of existing survey data with all identifying information removed. The study was approved by the ORC Macro Research Ethics Committee as well as Ethiopian Science and Technology Agency. Prior to the actual interview, each woman was asked if she agreed to participate in the study. The GIS data were obtained through direct review and approval from Measure DHS. Informed consent was obtained from the participants, their guardian or household heads.

## Results

### Socio-demographic characteristics

The mean age of the respondents was 30 years (SD ± 8.3). Forty-one percent of the respondents were within the age range of 25–34 years whereas 33.4% were in the range of 35–49 years. Over 65% of the respondents had no education, while 28% had primary education. About 41% of the respondents percent fell in the poor wealth quintile, 20% in middle, and 39% were classified in the richest quintile. Forty four percent of the respondents were followers of the Orthodox Christian faith, 31% were Muslim, 23% were Protestant, and the remaining 3% were members of other religions (Table 1).

**Table 1:** Socio-demographic characteristics of married women 15–49 years of age, 2011

**Variables**	**Number**^**£**^	**Percent (%)**
**Education**		
No education	6735	65%
Primary	2862	28%
Secondary	377	4%
Higher	313	3%
**Wealth**		
Poor	4194	41%
Middle	2083	20%
Rich	4010	39%
**Age**		
15-24	2527	25%
25-34	4231	41%
35-49	3529	34%
**Religion**		
Orthodox	4490	44%
Catholic	113	1%
Protestant	2319	23%
Muslim	3187	31%
Others	168	2%

^**£**^Total numbers are based on survey weighted figures.

### Modern contraceptive prevalence rate

**Figure 1:**
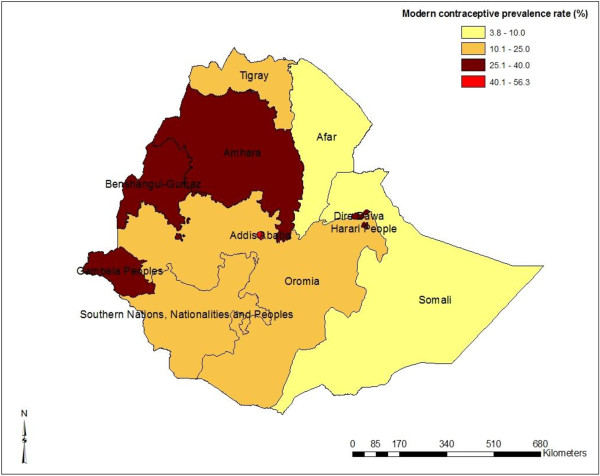
Map of regional modern contraceptive use among married women in Ethiopia, 2011.

Modern contraceptive prevalence rate was found to be 27.3% (urban 49.5%, rural 22.5%). There is variation in contraceptive prevalence rates across the country’s regions. The highest contraceptive prevalence rate was reported in Addis Ababa (56.3%). The modern contraceptive prevalence rates are presented in Table 2 and Figure 1.

**Table 2:** Prevalence of modern contraceptive use in currently married women by basic background characteristics, 2011

**Background characteristics**	**Total (n = 10287**^**£**^**)**	**Use of modern contraception (95% CI)**
**Region**		
Tigray	620	21.2 (18.08–24.32)
Affar	104	9.1 (3.60–14.60)
Amhara	2775	33.0 (31.50–34.50)
Oromiya	3961	24.9 (23.84–25.96)
Somali	232	3.8 (1.37–6.23)
Benishangul- Gumuz	124	26.3 (18.6–34.00)
SNNP	2022	24.7 (23.01–26, 39)
Gambela	41	33.2 (18.81–47.59)
Harari	28	31.5 (14.32–48.68)
Addis Ababa	342	56.3 (51.13–61.47)
Dire Dawa	38	31.7 (16.93–46.47)
**Residence**		
Urban	1843	49.5 (47.43–51.57)
Rural	8444	22.5 (22.12–22.88)

^**£**^Total numbers are based on survey weighted figures.

### Determinants of modern contraceptive use among married women

Wealthy women had two times higher odds of using modern contraceptives than poor married women. Married women who lived in rural areas had 30% lower odds of using modern contraceptives than urban married women. Educated women had better odds of using modern contraceptive methods than uneducated married women. Age had an inverse association with use of modern contraceptive methods. Older married women had lower odds of using modern contraceptive methods than younger married women. Muslim married women had 30% lesser odds of using modern contraceptive methods than Christians. Women who had worked or been employed had a 30% lower odds of using modern contraceptives compared to married women who had no employment history (Table 3).

**Table 3:** Socio-economic determinants for married women to use modern contraceptive methods, 2011

**Socio-economic variables**	**Total weighted (n = 10287)**	**Crude OR 95% CI**	**AOR 95% CI**
**Wealth**			
Poor	4194	1.0	1.0
Middle	2083	1.5 (1.3–1.7)	1.4 (1.1–1.8)
Wealthy	4010	3.1 (2.8–3.5)	1.9 (1.5–2.4)
**Residence**			
Urban	1843	1.0	1.0
Rural	8444	0.3 (0.3–0.3)	0.7 (0.5–0.9)
**Education**			
No education	6735	1.0	1.0
Primary	2862	1.8 (1.7–2.0)	1.3 (1.0–1.6)
Secondary	377	4.1 (3.3–5.1)	1.4 (1.0–2.1)
Higher	313	4.8 (3.8–6.0)	1.2 (0.7–1.9)
**Age**			
15-24	2527	1.0	1.0
25-34	4231	1.0 (0.9–1.1)	0.7 (0.6–0.9)
35-49	3529	0.7 (0.6–0.7)	0.5 (0.4–0.7)
**Religion**			
Christian	6922	1.0	1.0
Muslim	3187	0.5 (0.4–0.6)	0.7 (0.6–0.8)
Others	169	0.2 (0.1–0.4)	1.0 (0.5–2.0)
**Occupation**			
Have no work	4473	1.0	1.0
Have any type of work	5725	1.3 (1.2–1.5)	1.30 (1.1–1.6)

*OR* Odds Ratio, *AOR* Adjusted Odds Ratio, *CI* Confidence Interval.

The number of living children a woman had was significantly associated with use of modern contraceptive methods. A woman who had at least one child had higher odds of using modern contraceptives than a woman who had no children. Women who had polygamous marriage were by half less likely to use modern contraceptive methods than women in monogamous marriage. Child mortality had a significant inverse relation with use of modern contraceptive methods. Married women who had experience of child mortality were less likely to use modern contraceptive methods (P < 0.001) (Table 4).

**Table 4:** Effect of family background on use of modern contraceptives among married women, DHS 2011

**Variables**	**Total weighted (n = 10287)**	**Crude OR 95% CI**	**AOR 95% CI**
**Number of living children**			
0	1018	1.0	1.0
1-4	6002	1.7 (1.5–2.0)	2.0 (1.4–2.7)
1-8	2985	1.1 (0.9–1.3)	2.2 (1.5–3.3)
≥9	282	0.9 (0.7–1.3)	3.1 (1.6–6.1)
**Family size**			
1-4	3337	1.0	1.0
5-8	5879	0.8 (0.8–0.9)	1.2 (1.0–1.5)
≥9	1071	0.6 (0.5–0.7)	0.9 (0.6–1.3)
**Marital type**			
Monogamous	9147	1.0	1.0
Polygamous	1073	0.3 (0.3–0.4)	0.5 (0.3–0.7)
**Child mortality**			
Not experience	6668	1.0	1.0
One died	1994	0.6 (0.5–0.6)	0.6 (0.4–0.8)
≥ 2 died	1625	0.4 (0.4–0.5)	0.7 (0.5–0.9)
**Partner education**			
No education	5022	1.0	1.0
Primary	4062	1.6 (1.5–1.8)	1.0 (0.8–1.2)
Secondary	604	2.7 (2.3–3.2)	1.0 (0.7–1.4)
Higher	514	4.0 (3.3–4.9)	1.1 (0.7–1.6)

*OR* Odds Ratio, *AOR* Adjusted Odds Ratio, *CI* Confidence Interval.

Married women who attended community conversation programs and who were visited by health workers were significantly more likely to use modern contraceptives over their counterparts (Table 5).

**Table 5:** Effect of exposure to health services to use modern contraceptive methods for married women, 2011

**Exposure variables**	**Total weighted (n = 10287)**	**Crude OR 95% CI**	**AOR 95% CI**
**Listening radio**			
No	7107	1.0	1.0
Yes	3179	1.9 (1.7–2.1)	1.2 (1.0–1.4)
**Watching TV**			
No	8824	1.0	1.0
Yes	1462	3.0 (2.7–3.3)	1.3 (1.0–1.6)
**Reading newsletter**			
No	9797	1.0	1.0
Yes	487	2.7 (2.2–3.2)	0.9 (0.7–1.2)
**Attending community conversation program**			
Never attended	1505	1.0	1.0
Attended for 3 months	623	1.8 (1.5–2.2)	1.7 (1.4–2.1)
4-11 months ago	348	1.6 (1.3–2.1)	1.6 (1.2–2.0)
12 months and more	399	1.1 (0.9–1.4)	1.0 (0.7–1.3)
**Visited by health workers**			
No	8292	1.0	1.0
Yes	1988	1.4 (1.3–1.6)	1.2 (1.0–1.5)
**Visit health facility**			
No	6267	1.0	1.0
Yes	4013	2.0 (1.8–2.2)	1.2 (1.0–1.4)

*OR* Odds Ratio, *AOR* Adjusted Odds Ratio, *CI* Confidence Interval.

### Geographical variation in modern contraception use

**Figure 2:**
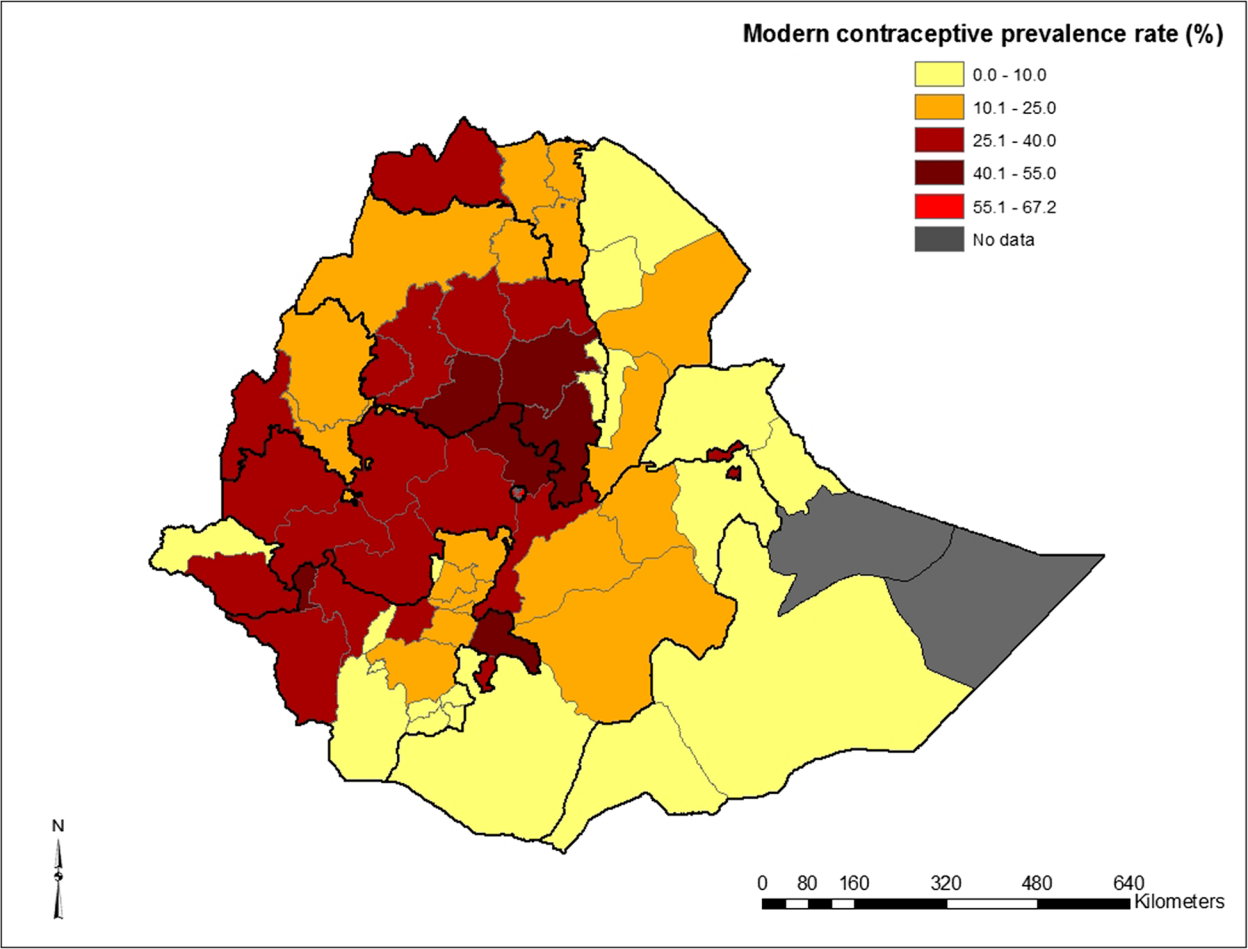
Map of zonal modern contraceptive use among married women in Ethiopia, 2011.

**Figure 3:**
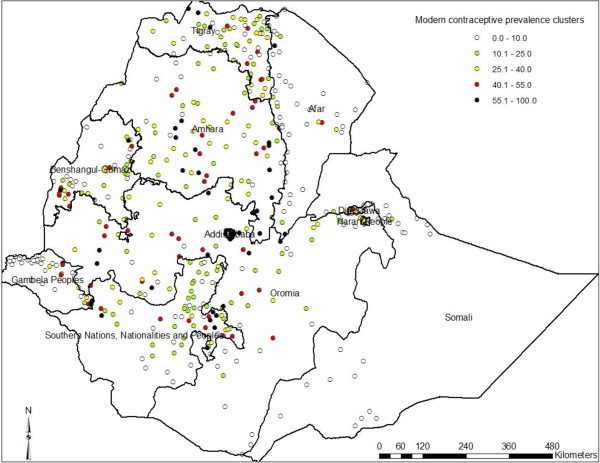
Spatial distribution of modern contraceptive use among married women in Ethiopia, 2011.

**Figure 4:**
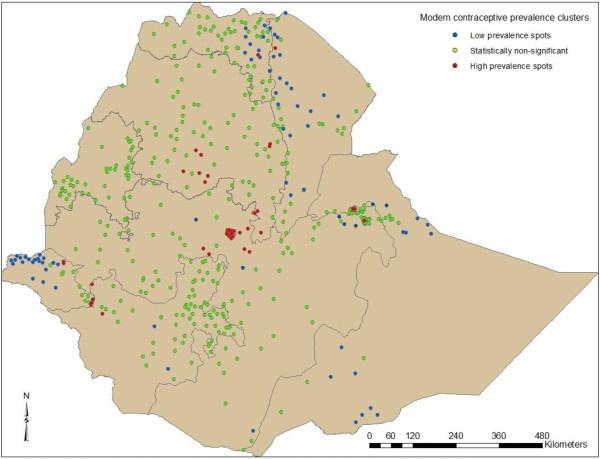
High and low prevalence clusters of modern contraceptive use among married women in Ethiopia, 2011.

Figure 1 and Table 6 show the regional variation in modern contraception prevalence rate where Addis Ababa, Amhara and some parts of Gambela and Benshangul Gumuz regions have high contraceptive prevalence. When the results were sub-divided by zone (Figure 2), the central and southwestern parts of the country had high prevalence of modern contraceptive use. The eastern and southern part of the country had lower prevalence of modern contraception use (Figure 3). The Getis-Ord G-statistic for spatial clustering of the use of modern contraception prevalence was significant for clusters with positive Z-scores (high prevalence spots) and with negative Z-scores (low prevalence spots) while most clusters returned values that suggest non-significant clustering (Figure 4). Most of the clusters of high prevalence were located in Addis Ababa region while most of clusters of low prevalence were in Affar, Somali and some parts of Gambela region (Figure 4).

**Table 6:** Trends in modern contraceptive use overtime and levels of unmet need in Ethiopian married women from 2000–2011 by region and residence

**Region**	**Percent of any modern contraceptive use**			**Percent of total unmet need for family planning**		
	**2000**	**2005**	**2011**	**2000**	**2005**	**2011**
Tigray	9.3	16.2	21.2	28.0	24.1	22.0
Affar	7.4	6	9.1	12.3	13.4	16.0
Amhara	6.6	15.7	33.0	40.9	29.7	22.1
Oromiya	4.3	12.9	24.9	36.4	41.4	29.9
Somali	2.4	2.7	3.8	14.3	11.6	24.0
Benishangul- Gumuz	8.5	10.4	26.3	31.9	29.7	24.5
SNNPR	5.0	11.4	24.7	35.5	37.4	25.0
Gambela	12.3	15.8	33.2	34.4	23.5	18.8
Harari	19.0	29.1	31.5	30.1	22.4	24.1
Addis Ababa	34.3	45.2	56.3	19.2	10.3	10.6
Dire Dawa	23.5	31.5	31.7	24.5	14.8	21.3
**Residence**						
Urban	28	42.2	49.5	25.0	17	15.0
Rural	3.0	10.6	22.5	37.3	35.8	27.5
Total	6.3	13.9	27.3	35.5	33.8	25.3

## Discussion

### Summary of the finings

In Ethiopia, 27.3% of married women in reproductive age group use modern contraceptive with wide urban rural variations (49.5% versus 22.5%). There is also variation in modern contraceptive prevalence rates across the country’s regions. Highest contraceptive prevalence rate was reported in Addis Ababa, Dire Dawa, Harari, Amhara and Gambela whereas comparatively low prevalence was reported in Affar and Somali regions. High prevalence spots were detected in the central highlands, in the other hand low prevalence spots were detected in the in the western, eastern and northeastern part of the country.

Richest wealth quintiles, higher level of education, being employed, higher number of living children, being in a monogamous relationship, attending community conversation, being visited by health worker at home increase the likelihood of using modern contraption. While living in rural areas, older age, being in polygamous relationship, and witnessing one’s own child’s death were found negatively influence modern contractive use. Modern contraceptive use also varies by religion.

### Strengths and limitations of the study

This study is based on a nationally representative data and used multivariate analysis to identify factors affecting the use of modern contraception in Ethiopia. The strength of the study is it used GPS data to assess the geographical distribution of the use of modern contraceptive use at region and zone level in Ethiopia. Furthermore the study identified high prevalence and low prevalence spots which inform the areas which needs priority. The health services coverage is very high in Ethiopia which decreases the variability in the health system which could have confounded the analysis.

There are limitations to the current study. The study focused mostly on individual and few contextual risk factors, it has not addressed wider social and cultural environment in which the outcomes occur. Although we have included many factors the list is not exhaustive. We have not included female autonomy [15,16], attitudes toward health service use [17], community fertility norms [15], community level approval of family planning [15], transport infrastructure [18,19], road access and distance from health facility [16,19], which were found to affect the use of contraction in previous studies. Future analysis should put greater emphasis on the use of community-level data. The current analysis included women of reproductive age who are married or in a union. Women who are sexually active but not married or in a union may have different contextual factors than married women and future studies should consider this. This study relied solely on quantitative data, and it is important that a better understanding of the effects of specific socialcultural factors that might underlie the effect of variables such as religions on modern contraceptive use are explored through future qualitative study.

### Implication of the finings

The high use of modern contraceptive prevalence rate found in urban over rural areas is consistent with the findings of a study conducted in southern Ethiopia [20]. This might be related to availability of contraceptive services, education, and wealth. Similarly, a study conducted in northern Ethiopia [21] also found that urban women had more access to health services than rural women. Findings from across the developing world showed that the better educated a women is, the more likely she is to use contraception [22,23]. Our analysis also shows that women’ educational status had a positive influence on modern contraceptive use. The influence of household wealth status and women’s employment status on contraceptive use was consistent with previous studies elsewhere [23,24].

In our analysis, younger women were more likely to used modern contraception than older women. This is an encouraging result which has implications on promising future trends of family planning utilization. The number of living children was also a factor influencing the use of modern contraception. Those women with more living children were more likely to use contraception. This suggests that contraception is adopted by high-parity women who wanted to cease childbearing [23]. This relationship has been described in other countries and may be linked to the desire for limiting and/or spacing childbirth by women [23,25-27]. Women who were in polygamous relationship were less likely to use contraception than those in monogamous relationship. This might be due to the nature of the relationships, where there may be competition for more children among women with the same husband.

Our findings revealed that women who had attended community conversation, and who were visited by a health provider were more likely to use modern contraceptives than their counterparts. A study conducted in Ethiopia showed that if respondents had visited a health clinic and received family planning advice or services, there was a significant association with the use of modern contraceptives [28]. Another study, [29] revealed that the use of family planning methods was found to be positively correlated with women’s exposure to information on family planning methods in television, radio, or newspapers. In our analysis there was no association with exposure to mass media and visiting health facility. Rather attending community conversion sessions and being visited by health provider were factors that contributed to the use of modern contraception. These implies that in the Ethiopian context individualized counseling approaches and community conversations are better alternatives than mass media to deal with concerns of women and increase use of family planning methods. Although family size and partners’ education were found to be factors affecting use of modern contraception in other studies, these factors did not independently predict the use of modern contraception in our analysis.

The spatial distribution of modern contraceptive use among married women in our study revealed that there are significant geographical variations among regions in Ethiopia. Particularly the regions of Affar and Somali have lower prevalence rates compared to other regions. The populations of the two regions are pastoralist characterized by seasonal mobility. The spatial distribution of zonal-level and cluster level prevalence of modern contraceptive use highlights the extent of disparity among zones and districts across Ethiopia. The low levels of contraceptive use in the Affar and Somali regions suggest that factors common to these regions may underlie the spatial patterns observed. The relative underdevelopment and low urbanization may contribute to the low contraceptive use.

The findings of this study are important for use by program managers involved in family planning in Ethiopia. Our findings of the ways in which aspects of the individual and household factors influence a woman’s use of modern contraception can be used by family planning program managers to shape the development of family planning provision and promotion programs. Health promotion can use community conversation which involves community wide discussion to address cultural beliefs and customs to increase approval of family planning. In addition, the spatial distribution using low and high prevalence clusters can be used to identify areas where existing contraceptive use is above or below expectations. Areas with higher than expected use may be examples of good practice that providers and policymakers could learn from to improve policy and practice, and areas of lower use could be targeted for future interventions. The importance of socioeconomic factors also lends support to development policies that removal of economic barriers to service use.

The geographical variation of the conceptive use needs furthers research, particular identifying the contextual factors contributing to the uptake of contraception in high prevalence clusters and the low prevalence clusters.

## Conclusions

The results highlight how individual factors influence ones use of modern contraception. The study particularly identified how socioeconomic status of women and number of children affects the use of contraception. There is evidence of wide geographical variation in modern contraceptive use in Ethiopia. Low prevalence clusters were located in Affar, Somali and some parts of Gambela Regional State of Ethiopia. The findings have several implications: first, providing employment and educational opportunities for women are important to increase uptake of contraception. Second community conversion and individuals counseling through home visit could help to address concerns and increase contraceptive utilization. Third, the three low-use regions should be targeted for scaling up and tailored services to the life styles of the population of the regions.

## Competing interests

The authors declare that they have no competing interests.

## Authors’ contributions

YL and KD conceived the study. YL analyzed the data. YL, HT and KD drafted the manuscript and reviewed the article. AAR, SB extensively reviewed the article. All authors read and approved the final manuscript.
